# The Contribution of Adverse Childhood Experiences and Neighborhood Characteristics on Outcomes Experienced by Urban Dwelling Black Men After Serious Traumatic Injury

**DOI:** 10.1007/s11524-024-00956-7

**Published:** 2025-01-22

**Authors:** Therese S. Richmond, Ryan Quinn, Anna Duan, Christopher N. Morrison, Nancy Kassam-Adams, Augustine Cassis Obeng Boateng, Sara F. Jacoby

**Affiliations:** 1 School of Nursing, University of Pennsylvania, 418 Curie Blvd, Fagin Hall 330, Philadelphia, PA 19104 USA; 2 School of Nursing, University of Pennsylvania, 418 Curie Blvd, Fagin Hall, Philadelphia, PA 19104 USA; 3 Penn Injury Science Center, University of Pennsylvania, Philadelphia, USA; 4 Weitzman School of Design, University of Pennsylvania, Philadelphia, USA; 5 School of Public Health, Columbia University, New York, NY USA; 6 School of Public Health and Preventive Medicine, Monash University, Melbourne, Australia; 7 Center for Injury Research & Prevention, Children’s Hospital of Philadelphia, Philadelphia, USA

**Keywords:** Injury, Depression, Posttraumatic stress disorder, Outcomes, Neighborhood, Adverse childhood experiences

## Abstract

Depression and post-traumatic stress disorder (PTSD) are serious consequences of physical injuries. Stress associated with living in urban neighborhoods with socioecological disadvantages and the cumulative burdens of adverse childhood experiences (ACEs) can lead to poorer psychological outcomes. Limited research has explored how ACEs and socioecological environmental exposures in childhood and adulthood, together, impact post-injury outcomes. This study assessed the relative contributions of ACEs and neighborhood exposures during childhood and adulthood on post-injury outcomes among Black men in Philadelphia. We used data from a prospective cohort of 414 Black men from the Philadelphia region, aged ≥ 18 years, who sustained acute physical injuries requiring hospitalization. Primary outcomes were post-injury PTSD and depression. Secondary outcomes were sleep quality, self-reported health status, changes in substance use, and return to work. The study used perceived and objective measures of neighborhood characteristics and self-reported ACEs to model their relative impact on outcomes 3 months after hospital discharge. Higher levels of ACEs and higher perceived neighborhood disorder during childhood and adulthood were significant predictors of PTSD and depression symptom severity. Perceived neighborhood disorder contributed to sleep disturbances and decline in post-injury health. Census/administrative objective measures of neighborhood disadvantage did not show consistent associations with post-injury outcomes. Findings suggest that both ACEs and subjective perception of neighborhood environments are critical factors influencing post-injury recovery in urban Black men. Interventions to improve post-injury outcomes should consider preventing ACEs and addressing the tangible conditions of neighborhoods and residents’ perceptions of their surroundings to promote health equity and injury recovery.

## Introduction

Place-based risks for traumatic injuries and exposure to socioecological disadvantage are hypothesized etiologies of racial disparities in injury recovery outcomes in US urban environments [1, 2]. Previous research has demonstrated that 45% of seriously injured Black men residing in the greater Philadelphia region experienced post-traumatic stress disorder (PTSD) and/or depression in the aftermath of serious physical injuries [3]. Black men injured in urban environments like Philadelphia often experience these kinds of post-injury sequelae in the context of other exposures associated with PTSD and depression like systemic racism, economic disenfranchisement, and disproportionate exposure to community violence, all of which significantly can influence their post-injury health trajectories [4]. However, there are other critical life course exposures such as adverse childhood experiences (ACEs) that may shape responses to stressful experiences such as serious injuries which make injury recovery even more complex and challenging.

Studies have demonstrated, independently, that the stress associated with living in urban neighborhoods with socioecological disadvantages, and the cumulative burdens of ACEs, can lead to poorer injury outcomes [5, 6]. However, there is limited research exploring how ACEs and socioecological environmental exposures in childhood and adulthood, together, impact injury outcomes. Thus, the purpose of this study was to determine the relative contribution of ACEs and the impact of neighborhood exposures in both childhood and adulthood on post-injury outcomes in Black men residing in the greater Philadelphia region. The primary aim was to examine the severity of PTSD and depression after injury. The secondary aims were to explore indicators inter-related with these key psychological outcomes including sleep quality, self-appraisal of post-injury health status, changes in substance use, and return to work.

This study looks across the life course and at the interplay between socioecological influences on injury risks and recovery within the home and in the broader neighborhood in which the home is located. ACEs capture exposures that typically occur within a residence, home, and family environment. CDC and Kaiser-Permanente’s sentinel ACE study demonstrated the profound pervasiveness of ACEs in the US population. These insights were derived from research on a sample of predominately White, middle-class individuals [7]. Subsequent research has documented significantly higher prevalence of at least one (“some exposure”) and more than four (“severe exposure”) ACEs in low-income and minority populations when compared to the general population [8].

The relative prevalence of ACEs in populations has been shown to have significant relationships with health impacting outcomes and exposures related to injury risks and outcomes. In a meta-analysis, when compared to adults who report no ACEs, those who report more than four ACEs have 7.5 times the odds (95% CI 5.6–10.1) of violence victimization and 8.1 the odds (95% CI 5.9–11.2) of violence perpetration [8–10]. ACEs have the potential to worsen the impact of an injury; for those who have experienced abuse and household dysfunction in childhood, major stressors in adult life such as serious traumatic injury can worsen unhealthy stress responses, mental health problems, use of alcohol and illicit substances, and violent behaviors [9, 11, 12].

Outside of the home, broader neighborhood exposures exert substantial effects on health and safety [13] and can worsen recovery after injury [14]. Exposure to neighborhood disadvantage, specifically, predicts poorer mental health in residents [15]. Conversely, community factors that encourage social cohesion and relationships with committed adults can be protective factors for safety and well-being conferred through a neighborhood environment [16, 17]. Moreover, the impact of neighborhood environmental effects begins early in the life course. A 2018 review of the evidence on neighborhoods and child development emphasized current gaps in this area and the need to study family environments in relationship to the people, resources, and opportunities within their lived environment [18].

Currently, there is insufficient evidence to explain the relationships between socioecological contexts in which ACEs arise and injury and injury’s long-term sequelae. Addressing this gap empirically is critical for developing interventions that address post-injury symptomology while also providing new evidence to guide injury prevention. The complexity of the health effects of ACEs may also need to be understood within the context of structural policies and processes that affect the neighborhoods in which people live and which shape their lived experiences [19]. One could posit that individuals who experienced ACEs and resided in neighborhoods with socioecological disadvantages would be at higher risk for poor injury outcomes and sequelae than those who had not experienced ACEs but resided in advantaged neighborhoods. This study positions ACEs within the broader context of socioecological determinants of injury and health with a goal of informing practice and policy. Moreover, responsibility for early childhood adversity is often relegated solely to parents and factors within the home and family unit. This work expands the narrative to include the broader conditions in which ACEs arise and extends the range of possible interventions which can impact injury outcomes along with a wide spectrum of health and social outcomes [19].

## Methods

This study was a secondary analysis of data collected for a prospective cohort study of psychological outcomes experienced by injured Black men recruited in Philadelphia, PA. The study design was guided by the socioecological model recognizing that the recovery experiences of injured individuals are affected by a range of social influences nested within complex environmental contexts. Eligibility for the cohort study was limited to men who self-identified as Black, were 18 years or older, could provide informed consent, and resided in the greater Philadelphia region. Exclusion criteria included pre-existing cognitive dysfunction or injury that impaired the ability to provide consent and participate in interviews; active psychotic disorder; suicide attempt; or being under professional care for treatment of depression or PTSD at the time of injury. Data were collected from January of 2013 through October of 2017.

Philadelphia is an ideal setting for this analysis. It is an urban environment with 1.6 million residents, with substantial racial, ethnic, and economic diversity [20]. With a rich history, multiple civic, educational, and healthcare institutions, it remains the poorest of the 10 largest US cities where over 26% of residents (and 37% of children) live below the federal poverty line and where 31% of Black and 41% of Hispanic residents live below the federal poverty line compared to 18% of White residents [21]. The city’s geography also illustrates high racial and economic segregation associated with physically and mentally impactful health exposures within its varied built environment and social environments [22].

The original study cohort consisted of 623 adult Black men who sustained an acute physical injury of any mechanism that required hospitalization of more than 24 h in a regional resource (level 1) trauma center in an academic medical center, located in urban Philadelphia. In-hospital baseline, interviews took place when participants were medically stable. Injury and medical data were obtained from medical records and the trauma registry. Follow-up data were obtained from 500 of the enrolled participants at 3 months post-discharge in participants’ homes. Interviews were conducted in English. Of these, participants for this present study were excluded if they were born prior to 1945 (*n* = 15) due to the challenge of obtaining childhood neighborhood data from historical census records and if they did not provide ACE survey responses (*n* = 71), generating a final sample of 414. Participant characteristics were compared between those included (*n* = 414) and excluded (*n* = 209) from the present analysis to assess for any systematic differences using two sample *t*-tests and chi square tests for continuous and categorical measures, respectively.

### Instruments

#### Predictor Measures: ACEs

ACEs were collected at the baseline interview. Dichotomous (yes/no) items were grounded with the stem “While you were growing up during your first 18 years of life…” identical to that of the original Felitti et al. ACE study [7]. The stem “did a parent or other adult in your household…” served as the anchor to ask about psychological, physical, and sexual adverse experiences. The remaining ACE items captured household: substance use, mental illness, violent treatment of mothers, and criminal behavior. The possible exposure score ranges from 0 (unexposed) to 7 (exposed to all categories).

#### Predictor Measures: Neighborhood Characteristics

Perceived levels of neighborhood order/disorder were obtained using the Neighborhood Environment Scale (NES) which has demonstrated face validity in the context of Philadelphia [23], with high internal consistency (e.g., KR20 = 0.85) [24]; participants answer yes/no to questions about conditions in the area they considered to be their neighborhood (e.g., feeling safe; observations of social disorder); the total score ranges from 0 to 18 [24]. Participants completed the NES reflecting their neighborhood at the time of injury and in recall of their neighborhood at the age of 13.

Objective exposure to neighborhood disadvantage at the time of injury was determined by geocoding participants’ residential addresses to census tract-level data. Adult neighborhood exposures were based on residence at the time of injury at the census tract-level data with socioecological data merged from the American Community Survey Application Programming Interface (API) provided by the US Census Bureau. Census tract level data were standardized across the three counties of residence from which participants were recruited. Following an exploratory analysis, a summative neighborhood disadvantage index was calculated by indexing measures used in other empiric research to determine census tract level concentrated disadvantage and the intracity social and economic inequity [25], percentage of adults without a high school education, percentage of children living in single parent families, percentage of individuals above 16 years who are unemployed, inverse of the standard racialized index of concentration at the extremes (percentage of the population that is Black relative to the percentage of the population that is White) which is used to measure relative intra-city racial segregation and inequality [26], the prevalence of property and violent crime, the inverse of the distance to the nearest primary care facility, and the inverse of the distance to the nearest hospital for each participant. The summed measures exhibited good internal consistency (Cronbach’s alpha = 0.77).

Objective exposure to neighborhood disadvantage during childhood/early adolescence was determined by geocoding participants’ residential addresses at the age of 13 to historic decennial census tract data from the National Geographic Information System provided by ipums.org. First, historic census tract level data were obtained for all participants’ childhood address data between 1960 and 1990. Next, neighborhood characteristics were standardized, within decade, among greater Philadelphia region census tracts containing participants’ childhood addresses in the sample. These data were merged with participant data by census tract and decade. Standardized indicators of neighborhood social disadvantage in childhood were constructed per census tract and decade using percentage of the population who were Black, percentage unemployed, and percentage who had not achieved high school graduation. These measures are partial components of standard indices of concentrated disadvantage included in statistical modeling using historic census due to consistent availability of additional components across the study period.

#### Outcomes: Psychological Outcomes

PTSD symptom severity was measured with the PTSD Checklist-5 (PCL-5), a validated 20-item self-report instrument. The PCL-5 assesses current symptoms of PTSD in relation to the injury event leading to hospitalization and has been widely used in racially diverse populations. It has shown strong internal consistency (Cronbach’s alpha = 0.94), test–retest reliability (r = 0.82), and convergent (rs = 0.74 to 0.85) and discriminant (rs = 0.31 to 0.60) validity [27]. Scores range from 0 to 80 with higher scores indicating more severe symptoms and ≥ 34 consistent with PTSD diagnosis. Depression symptom severity was measured with the Quick Inventory of Depressive Symptoms-self report (QIDS-SR_16_) with high internal consistency (Cronbach’s alpha = 0.92) and scores highly correlated with the common clinically applied Hamilton Depression Rating Scale (*r* = 0.86) [28]. QIDS scores range from 0 to 27 (higher scores indicating more severe symptoms); a score of ≥ 11 is consistent with meeting diagnostic criteria for major depression.

#### Outcomes: Health

Return to pre-injury health was measured on a Likert-type scale in response to the question “Compared to before your injury, how would you rate your health in general now?” (1 = much worse to 5 = much better). Substance use was measured with the question “What is the change in substance use since your injury?” (more/same/less). Sleep disturbance was measured with the 8-item PROMIS™ Sleep Disturbance short form asking about sleep in the previous 7 days [29]. Scores range from 8 to 40 with higher scores reflecting more disturbance. Validity and reliability are well established. Responses were dichotomized as more vs. same/less. Return to work was measured by the question “Are you back to work that you were doing before your injury?” (yes/no).

#### Co-variates: Injury Characteristics and Pre-Injury Health

Injury intent was dichotomized as intentional (e.g., gunshot wound, stabbing, blunt assault) or unintentional (e.g., motor vehicle crash, pedestrian injury, fall). Injury severity was measured using the Injury Severity Score (ISS), an anatomical measure that provides a numerical score of 1 (least severe) to 75 (most severe) to summarize the severity of multiple injuries across body systems, and dichotomized as severe (≥ 9) and not severe (< 9) [30, 31]. Two indicators of pre-injury health status were adapted from the 4-item Health-Related Quality of Life (HRQOL-4) scale used in the Behavioral Risk Factor Surveillance System (BRFSS) questionnaire and were collected by self-report: number of days of poor physical health and number of days of poor mental health in the 30 days before injury, dichotomized as 5 or more days and less than 5 days [32].

### Statistical Analysis

Participant characteristics and outcome data were summarized using means and standard deviations or frequencies and percentages for continuous or categorical measures, respectively. Variable distributions were assessed using histograms and quantile plots and tested for normality using Shapiro–Wilk tests. Bivariate plots assessed potential non-linear relationships between predictor and outcome variables. Participant characteristics were compared between those who completed the 3-month follow-up and those who did not.

The contribution of ACEs, childhood neighborhood characteristics, and adult neighborhood characteristics was assessed for continuous outcomes using linear mixed effects models. For categorical outcomes, generalized estimating equation models were employed with an exchangeable covariance matrix to account for anticipated correlation of errors within participants’ adult census tract. Models were adjusted for injury intent, injury severity, and pre-injury physical and mental health status. Analysis was conducted using SAS 9.4. An alpha level of 0.05 was used to determine statistical significance.

Due to systematically missing data from the historic census records, the primary analysis could not include predictors representing objective measures of childhood exposure to neighborhood disadvantage for the entirety of the sample. However, sensitivity analyses were conducted which included objective childhood exposure to neighborhood disadvantage predictors in the subsample of participants for whom these data could be derived. Participant characteristics were compared between those with missing and non-missing objective childhood neighborhood data. All models were adjusted for injury intent, injury severity, pre-injury physical health, and pre-injury mental health.

## Results

### Samples

The sample for the primary analysis comprised 414 men who self-identified as Black (4.1% also self-identified as Hispanic), with a mean age of 35.8 (SD 14.0), who were predominately single (62.8%), employed/student (56.8%), and who completed at least a high school education (68.3%). Over half (54.6%) were intentionally injured and 43.7% reported ≥ 3 ACEs (see Tables 1 and 2). The 414 men included in analyses were more frequently single (62.8% vs. 52.4%, *p* = 0.015) compared to participants excluded from analysis (due to missing ACE data, being born before 1945, or loss to follow-up at 3 months).

**Table 1:** Sample characteristics

*Variables*		*Full sample (n* = *414)*	*Sensitivity sample (n* = *297)*
Age	Mean (SD)	35.8 (14.0)	33.9 (13.6)
Hispanic	Yes	17 (4.1%)	13 (4.4%)
	No	393 (95.4%)	281 (95.3%)
	Unknown/declined response	2 (0.5%)	1 (0.3%)
Relationship status	Single (never been married)	260 (62.8%)	200 (67.3%)
	Married or living with a partner	136 (32.9%)	88 (29.6%)
	Divorced, separated, widowed	18 (4.3%)	9 (3.0%)
Current employment	Employed, student, retired	235 (56.8%)	173 (58.2%)
	Unemployed	179 (43.2%)	124 (41.8%)
Household income	< $20,000	159 (39.4%)	116 (39.9%)
	$20,000 to $39,999	73 (18.1%)	52 (17.9%)
	≥$40,000	50 (12.4%)	32 (11.0%)
	Unknown/declined response	122 (30.2%)	91 (31.3%)
Education	Less than high school	88 (21.3%)	58 (19.6%)
	Completed high school/GED	282 ( 68.3%)	211 (71.3%)
	Some/completed college	43 (10.4%)	27 (9.1%)
Has children	Yes	270 (65.2%)	187 (63.0%)
	No	144 (34.8%)	110 (37.0%)
Residence	Owns	75 (18.1%)	57 (19.2%)
	Rents	201 (48.6%)	138 (46.5%)
	Lives with family or friends	138 (33.3%)	102 (34.3%)
History of military service	Yes	27 (6.5%)	18 (6.1%)
	No	387 (93.5%)	279 (93.9%)

**Table 2:** Study variables

*Variables*		*Full sample (n* = *414)*	*Sensitivity sample (n* = *297)*
Total ACE categories reported	Mean (SD)	2.5 (2.0)	2.5 (2.0)
3 or more ACE categories reported	No	233 (56.3%)	165 (55.6%)
	Yes	181 (43.7%)	132 (44.4%)
Pre-injury poor physical health days	< 5 days	322 (77.8%)	231 (77.8%)
	≥ 5 days	92 (22.2%)	66 (22.2%)
Pre-injury poor mental health days	< 5 days	283 (68.4%)	201 (67.7%)
	≥ 5 days	131 (31.6%)	96 (32.3%)
NES: childhood perceived neighborhood disorder	Mean (SD)	8.7 (4.1)	9.1 (4.1)
NES: adulthood perceived neighborhood disorder	Mean (SD)	7.9 (4.3)	8.2 (4.3)
Adult neighborhood disadvantage index	Mean (SD)	5.2 (3.7)	5.2 (3.7)
QIDS score; depression severity	Mean (SD)	8.7 (5.7)	8.6 (5.7)
Screened positive for depression	No (≤ 10)	268 (64.9%)	201 (67.9%)
	Yes (> 10)	145 (35.1%)	95 (32.1%)
PCL-5 score PTSD severity	Mean (SD)	24.1 (19.0)	24.3 (19.1)
Post-injury returned to work	No	256 (61.8%)	182 (61.3%)
	Yes	158 (38.2%)	115 (38.7%)
Post-injury health	Same or better	209 (50.5%)	147 (49.5%)
	Somewhat or much worse	205 (49.5%)	150 (50.5%)
PROMIS sleep score: sleep disturbance	Mean (SD)	22.9 (10.1)	23.1 (10.3)

It was not possible to obtain objective childhood neighborhood data for 117 participants. The sample for the sensitivity analysis comprised 297 participants who were significantly older, perceived a more disordered adult and childhood environment, and more frequently married (*p* < 0.05) than the sample that comprised the primary analysis (see Tables 1 and 2).

### Primary Analysis

Fully adjusted models (Tables 3 and 4) showed that the number of ACEs reported (*B* = 0.93, *p* = 0.05), higher levels of perceived neighborhood disorder in adulthood and childhood (*B* = 0.67, *p* = 0.004 and *B* = 0.56, *p* = 0.01, respectively), intentional injury (*B* = 6.41, *p* < 0.001), more severe injury (*B* = 4.23, *p* = 0.02), and poorer pre-injury mental and physical health (*B* = 4.88, *p* = 0.02 and *B* = 4.30, *p* = 0.05, respectively) predicted PTSD symptom severity at the 3-month follow-up. Similarly, the number of reported ACEs (*B* = 0.33, *p* = 0.03), higher perceived adulthood neighborhood disorder (*B* = 0.18, *p* = 0.01), more severe injury (*B* = 1.4, *p* = 0.01), and poorer pre-injury mental and physical health (*B* = 1.76, *p* = 0.01 & 1.63, *p* = 0.02, respectively) predicted depression symptom severity at 3 months.

**Table 3:** Fully adjusted models for primary outcomes of Depression and PTSD symptom severity at 3 months post-injury

	*Full sample*			*Sensitivity sample*		
	***ß***	**SE**	***p***	***ß***	**SE**	***p***
PTSD (PCL-5 score)						
Number of ACEs	**0.93**	**0.48**	**0.05**	**1.53**	**0.57**	**0.01**
Adulthood NES score	**0.67**	**0.23**	**0.01**	**0.74**	**0.28**	**0.01**
Childhood NES score	**0.56**	**0.22**	**0.01**	0.22	0.27	0.42
Adult census tract neighborhood disadvantage index	0.09	0.23	0.69	0.21	0.27	0.44
Childhood census tract % Black population				1.24	1.39	0.37
Childhood census tract % without high school diploma				− 0.73	1.13	0.52
Childhood census track % unemployed				− 1.72	1.40	0.22
Intentional injury (versus unintentional)	**6.41**	**1.75**	**0.01**	**6.21**	**2.12**	**0.01**
Pre-injury poor physical health ≥ 5 days	**4.30**	**2.08**	**0.05**	**5.61**	**2.45**	**0.03**
Pre-injury poor mental health ≥ 5 days	**4.88**	**2.00**	**0.02**	4.04	2.31	0.09
Injury severity ≥ 9 (versus < 9)	**4.23**	**1.73**	**0.02**	2.98	2.04	0.15
Depression (QIDS score)						
Number of ACEs	**0.33**	**0.15**	**0.03**	**0.54**	**0.18**	**0.00**
Adulthood NES score	**0.18**	**0.07**	**0.01**	0.13	0.09	0.15
Childhood NES score	0.06	0.07	0.36	0.03	0.08	0.70
Adult census tract neighborhood disadvantage index	0.02	0.07	0.79	0.04	0.09	0.61
Childhood census tract % Black population				0.33	0.43	0.44
Childhood census tract % without high school diploma				− 0.26	0.35	0.46
Childhood census track % unemployed				− 0.33	0.43	0.44
Intentional injury (versus unintentional)	0.91	0.54	0.10	1.16	0.65	0.08
Pre-injury poor physical health ≥ 5 days	**1.63**	**0.65**	**0.02**	**2.35**	**0.75**	**0.00**
Pre-injury poor mental health ≥ 5 days	**1.75**	**0.61**	**0.01**	1.25	0.72	0.09
Injury severity ≥ 9 (versus < 9)	**1.38**	**0.53**	**0.01**	1.22	0.63	0.06

**Table 4:** Fully adjusted models for secondary outcomes of sleep disturbance, health appraisal, return to work, and substance use at 3 months post-injury

	*Full sample*			*Sensitivity sample*		
Sleep disturbance (PROMIS sleep score)	ß	SE	*p*	ß	SE	*p*
Number of ACEs	0.07	0.27	0.78	0.36	0.33	0.27
Adulthood NES score	**0.37**	**0.13**	**0.01**	**0.36**	**0.16**	**0.03**
Childhood NES score	0.17	0.13	0.17	0.07	0.16	0.67
Adulthood neighborhood disadvantage index	− 0.03	0.13	0.82	− 0.08	0.16	0.61
Childhood census tract % Black population				1.07	0.80	0.18
Childhood census tract % without high school diploma				− 0.82	0.66	0.22
Childhood census track % unemployed				− 0.67	0.81	0.41
Intentional injury (versus unintentional)	0.36	1.00	0.72	0.64	1.22	0.60
Pre-injury poor physical health ≥ 5 days	**3.13**	**1.19**	**0.01**	**4.57**	**1.42**	**0.01**
Pre-injury poor mental health ≥ 5 days	1.30	1.13	0.26	0.70	1.35	0.61
Injury severity ≥ 9 (versus < 9)	**2.00**	**0.98**	**0.05**	1.50	1.18	0.21
Worse post-injury health	OR	95%CI	*p*	OR	95%CI	*p*
Number of ACEs	**1.13**	**1.01–1.25**	**0.03**	**1.19**	**1.04–1.35**	**0.01**
Adulthood NES score	1.05	0.99–1.10	0.08	1.06	0.99–1.13	0.08
Childhood NES score	1.03	0.98–1.08	0.28	1.02	0.95–1.08	0.61
Adulthood neighborhood disadvantage index	1.01	0.95–1.07	0.69	1.00	0.92–1.08	0.97
Childhood census tract % without high school diploma				0.85	0.63–1.14	0.28
Childhood census tract % Black population				1.13	0.78–1.63	0.53
Childhood census track % unemployed				1.12	0.79– 1.6	0.52
Intentional Injury (versus unintentional)	1.09	0.73–1.63	0.67	0.98	0.58–1.66	0.98
Pre-injury poor physical health ≥ 5 days	**1.67**	**1.02–2.74**	**0.04**	**2.25**	**1.18–4.3**	**0.01**
Pre-injury poor mental health ≥ 5 days	1.01	0.64–1.62	0.95	0.97	0.56–1.69	0.91
Injury severity ≥ 9 (versus < 9)	**1.85**	**1.17–2.95**	**0.01**	**2.05**	**1.16–3.63**	**0.01**
Return to work	OR	95%CI	*p*	OR	95%CI	*p*
Number of ACEs	1.12	0.99–1.28	0.08	1.17	0.99–1.37	0.06
Adulthood NES score	1.03	0.97–1.08	0.33	1.05	0.98–1.13	0.17
Childhood NES score	0.97	0.92–1.03	0.31	0.93	0.87–0.99	0.30
Adulthood neighborhood disadvantage index	1.02	0.96–1.07	0.56	0.98	0.87–0.99	0.63
Childhood census tract % Black population				1.26	0.87–1.83	0.22
Childhood census tract % without high school diploma				**0.67**	**0.49–0.93**	**0.02**
Childhood census track % unemployed				1.01	0.76–1.36	0.93
Intentional Injury (versus unintentional)	0.66	0.42–1.04	0.07	0.64	0.31–1.11	0.11
Pre-injury poor physical health ≥ 5 days	**2.06**	**1.19–3.18**	**0.01**	**2.71**	**1.36–5.39**	**0.01**
Pre-injury poor mental health ≥ 5 days	0.82	0.50–1.34	0.42	0.77	0.34–1.37	0.37
Injury severity ≥ 9 (versus < 9)	**2.06**	**1.41–3.00**	**0.00**	**2.22**	**1.38–3.56**	**0.00**
Increased substance use	OR	95%CI	*p*	OR	95%CI	*p *
Number of ACEs	1.06	0.88–1.27	0.54	1.12	0.89–1.42	0.32
Adulthood NES score	1.06	0.96–1.17	0.26	1.05	0.93–1.19	0.41
Childhood NES score	1.07	0.96–1.20	0.22	1.11	0.97–1.27	0.13
Adulthood neighborhood disadvantage index	0.97	0.86–1.10	0.66	1.01	0.88–1.15	0.88
Childhood census tract % Black population				0.77	0.43–1.36	0.43
Childhood census tract % without high school diploma				**1.64**	**1.04–2.59**	**0.03**
Childhood census track % unemployed				0.99	0.46–2.11	0.98
Intentional injury (versus unintentional)	1.71	0.81–3.62	0.15	**3.14**	**1.29–7.63**	**0.01**
Pre-injury poor physical health ≥ 5 days	0.91	0.35–2.37	0.84	0.82	0.28–2.37	0.81
Pre-injury poor mental health ≥ 5 days	2.0	0.09–4.46	0.09	1.99	0.73–5.44	0.18
Injury severity ≥ 9 (versus < 9)	0.95	0.43–2.07	0.89	1.14	0.49–2.68	0.80

Post-injury sleep disturbances were significantly predicted by perceived neighborhood disorder in adulthood (*B* = 0.37, *p* = 0.005), poorer pre-injury physical health (*B* = 3.13,* p* = 0.01), and more severe injury (*B* = 2.00, *p* = 0.0465). For each additional ACE reported, the odds of participants reporting post-injury health decline increased by 1.13 times (OR = 1.13, 95% CI 1.01–1.25, *p* = 0.03) when compared to reporting post-injury health stability or improvement. Participants with poorer pre-injury physical health were estimated to have 1.67 times increased odds of reporting post-injury health decline (OR = 1.67,95% CI 1.02–2.74, *p* = 0.04), and those with a more severe injury (ISS ≥ 9) were estimated to have 1.8 times increased odds of reporting post-injury health decline (OR = 1.85, 95% CI 1.17–2.95, *p* = 0.01).

No predictor measures were significantly associated with the outcomes of return to work or an increase in post-injury substance use (alcohol/drugs).

### Sensitivity Analysis

We conducted sensitivity analyses for the subsample for whom objective childhood exposure to neighborhood disadvantage could be derived. Fully adjusted sensitivity models demonstrated that PTSD symptom severity was significantly predicted by number of ACEs (*B* = 1.53, *p* = 0.008), perceived adult neighborhood disorder (*B* = 0.74, *p* = 0.009), intentional injury (*B* = 6.21, *p* = 0.005), and poorer pre-injury physical health (*B* = 5.61, *p* = 0.03). Depression symptom severity was significantly predicted by the number of ACEs (*B* = 0.54, *p* = 0.003) and poorer pre-injury physical health (*B* = 2.35, *p* = 0.004).

Post-injury sleep disturbance was significantly predicted by perceived adult neighborhood disorder (*B* = 0.36, *p* = 0.03) and poorer pre-injury physical health (*B* = 4.57, *p* = 0.003). Higher level population education attainment in the census tract in which a participant lived in childhood and higher childhood perceived neighborhood disorder was associated with higher odds of returning to work (OR = 0.67, 95% CI 0.49–0.93, *p* = 0.02 and OR = 0.93, 95% CI 0.87–0.99, *p* = 0.03, respectively).

Participants with poorer pre-injury physical health had 2.7 times increased odds of not returning to work (OR 2.71, 95% CI 1.36–5.39, *p* = 0.005). Additionally, participants with more severe injury had 2.2 times increased odds of not returning to work (OR = 2.22, 95% CI 1.38–3.56, *p* = 0.001).

With each additional ACE reported, participants’ odds of reporting post-injury health decline increased (OR = 1.19, 95% CI 1.04–1.35, *p* = 0.008) compared to those reporting post-injury health as stable or improved. Further, participants with poorer pre-injury physical health had over twice the odds of reporting post-injury health decline (OR = 2.25, 95% CI 1.18–4.30, *p* = 0.01) and those with more severe injury had two times the odds of reporting post-injury health decline (OR = 2.05, 95% CI 1.16–3.63, *p* = 0.01).

Living in a census tract with a lower level of population education attainment in childhood was associated with greater odds of self-reported increase in post-injury substance use (OR = 1.64, 95% CI 1.04–2.59, *p* = 0.03), and participants with intentional injury had over three times higher odds of reporting increase in substance use post-injury (OR 3.14, 95% CI 1.29–7.63, *p* = 0.01).

## Discussion and Conclusion

This study evaluated the impact of ACEs and the characteristics of neighborhood social and physical environments in a cohort of urban dwelling Black men during childhood and adulthood, on post-injury outcomes including PTSD symptoms severity, depression symptom severity, return to health and work, and sleep disturbances. Our findings indicate that while ACEs were predictive of certain outcomes, they did not account for all. Specifically, a higher number of ACEs was associated with greater severity of post-injury depression symptoms and increased likelihood of reporting post-injury health decline. Contrary to existing evidence, exposure to neighborhood disadvantage, as measured by census tract level data, was not significantly associated with post-injury outcomes. This challenged our expectations and some well-established notions about the effects of objective neighborhood exposures, measured via census and other administrative data, on injury recovery and other health outcomes [33, 34].

In analyses that included census data on participants’ childhood neighborhood of residence, ACEs were again associated with some, but not all, post-injury outcomes. Specifically, ACEs were associated with the severity of PTSD and depression symptoms, as well as self-reported post-injury health decline. This suggests, as other studies have before, that childhood adversity, including ACEs and broader socioecological factors, plays a complex role in shaping long-term health outcomes. Previous research has connected ACEs with PTSD symptom severity in violence survivors [10], yet our primary models did not reflect this relationship, underscoring the potential cumulative impact of individual and neighborhood stressors over the life course.

One reason that objective neighborhood characteristics may not have shown significant associations with post-injury outcomes in our study is a lack of heterogeneity of participants’ neighborhood environments within the greater Philadelphia region, resulting in limited variation in the socioecological context of the census tracts studied. Philadelphia is and has been a highly racially segregated city where upward of 40% of the population is Black and a relatively high percentage of Black residents live in neighborhoods associated with socioecological disadvantage [35]. Moreover, census data rely on administratively defined boundaries, which may not align meaningful social exposures within the neighborhoods in which participants resided both at the time of injury and in childhood [15]. Alternatively, more precise methods of objectively measuring neighborhood conditions, such as direct observations of smaller-level geographies like city blocks, have been useful to examine a variety of health outcomes including post-injury outcomes like sleep quality [36].

A critical insight emerging from this study was the greater significance of perceived neighborhood assessments of order/disorder compared to objective assessments of neighborhood advantage/disadvantage. Perceived neighborhood disorder at the time of injury was significantly associated with post-injury PTSD and depression symptom severity, as well as sleep disturbances. A recent systematic review supports this finding linking neighborhood disorder with sleep disturbance across studies [37]. Additionally, perceived childhood neighborhood disorder was significantly associated with PTSD symptom severity after injury. While objective measures, from census data, provide a replicable and easily quantifiable view of neighborhood disadvantage, they may miss the nuanced and subjective experiences of individuals within these environments integral to the development of inter-related physical-psychosocial health outcomes. Perceived neighborhood disorder, for instance, may capture a range of psychosocial stressors such as fear of crime victimization, the lack of social cohesion, and degradation of the built environment all which may be felt by residents but not consistently reflected in census or other administrative data like local crime data. These subjective perceptions may also have more immediate or proximate impact on recovery and health outcomes in the months following a traumatic injury. In qualitative studies of urban dwelling Black men in the aftermath of traumatic injuries, individuals often express an increased sense of social isolation and fear of further injury based on neighborhood of residence [14]. Finally and more broadly, the significance of perception of neighborhood disorder aligns with growing evidence suggesting that how individuals perceive their environment can be as, if not more, influential on health outcomes than an environment’s objective characteristics [38].

The findings of this study must be interpreted in the context of its limitations. This is a secondary analysis of a longitudinal cohort study that was not designed for the aims of this research and relies on retrospective recall of ACEs and perceptions of childhood neighborhood characteristics. Members of the cohort, who vary in age, will have a varied length of time since their childhood which could impact the quality of this recall. The study includes an attenuated sample of the parent cohort due to some, albeit limited, missing data on primary predictor variables. Moreover, our analysis is of a cohort of urban dwelling Black men, a plurality of whom are lower income, single, injured intentionally and who had been exposed to at least 3 ACEs. This sample may not be representative of urban dwelling Black men who experience injuries, broadly. We were also bound by the operationalization of ACEs as collected for the parent cohort study. There may be more sensitive and specific ways to measures ACEs for use with urban dwelling Black men, including the Philadelphia ACE score, which is an expanded ACE index developed in Philadelphia which integrates extra-household exposures to community violence, peer victimization, and peer rejection [39]. Finally, we included ACEs as a cumulative exposure. Emerging evidence suggests the value of examining specific categories and clusters of ACEs for their effects on adverse health outcomes over time [40].

To our knowledge, this study is the first to examine the extent to which ACEs and neighborhood characteristics, across the life course, are associated with post-injury psychological outcomes in urban dwelling Black men who have experienced serious physical trauma. While the findings reveal a complex interplay between ACEs and neighborhood factors on post-injury outcomes, one of the most salient conclusions is that subjective perceptions of neighborhood environments appear to impart a more direct impact on post-injury recovery than objective measures of neighborhood disadvantage even when controlling for pre-injury health and injury characteristics. This highlights the need for strategies that not only address the physical and psychological aftermath of injury but also consider the meaning of the socioecological context in which individuals live and have lived.

Interventions that aim to improve post-injury outcomes and seek to address enduring racial disparities in injury outcomes might benefit from focus on the prevention of ACEs along with efforts to improve the tangible conditions of neighborhoods and the quality of residents’ experiences within their communities. The interrelationship between ACEs, neighborhood exposures, and injury outcomes illustrated in this work also highlights the potential of programmatic and policy interventions that attend to factors across the socioecological continuum. These foci include more traditional interventions such as prevention of ACEs and optimization of injury recovery designed for individuals and families. This research emphasizes the need to concurrently focus on overarching structural determinants of socioecological disadvantage such as disinvestment in the tangible conditions of specific neighborhoods, as a part of the necessary spectrum of approaches aimed to reduce risks for ACEs and improve outcomes after injury.

## Data Availability

The data analyzed in this study are not publicly available to protect participant privacy. However, certain data and additional details related to the study are available upon reasonable request.
